# 

**Published:** 1853-01

**Authors:** 


The anuual meeting of the Erie County Medical Society, will be held on the second Tuesday in January, at the American Hall, Buffalo, at eleven o'clock, A. M.

The members will recollect that the meeting is expected to possess unusual interest from the arrangements made by a committee to provide a dinner at the close of the session.

---

The regular term of Lectures at the Buffalo Medical College, will commence on Wednesday, the 5th of January. The Introductory Lecture will be delivered by Prof. Moore, at 2, P. M.

